# Anakinra: a silver lining in COVID-19?

**DOI:** 10.1186/s13054-020-03312-8

**Published:** 2020-10-06

**Authors:** Rahul Gupta

**Affiliations:** Independent Researcher, India

Dear Editor,

In the recent review article *Blocking IL-1 to prevent respiratory failure in COVID-19* by Dr. Veerdonk and Dr. Netea [1], they have implicated the importance IL-1 signaling in severe COVID-19 pathogenesis and proposed anakinra as a therapeutic intervention. I would like to humbly add some views to it; in severe COVID-19 pathology, higher amounts of microthrombi deposition can lead to the blockage in the capillaries and arteries culminating in hypoxic condition and ensuing necrosis-like events. In fact, autopsy studies have reported necrotic events of lung pneumocytes in severe COVID-19 patients [2]. Also, high serum levels of receptor-interacting protein kinase 3 (RIPK-3) in severe patients corroborate necroptosis too [3].

The damage-associated molecular pattern IL-1α (alarmin) liberated by necrotizing lung epithelial cells could be one of the initial cytokine produced during COVID-19 pathogenesis [4]. IL-1α by further engagement to IL-1R lead to the production of an array of chemokines and cytokines [4]. IL-1β, IL-6, TNF-α, GM-CSF, IL-17, CXC chemokines, CCL chemokines, etc. are some of the few upregulated cytokines/chemokines in severe COVID-19 responsible for exacerbating the lung pathophysiology by fueling up infiltration of macrophages/neutrophils, hypercoagulability, and fibrosis phenomena [1]. Interestingly, IL-17, IL-6, GM-CSF, HIF-α, and CXC chemokines are regulated by IL-1β. The hypercoagulability with thromboembolic indications in severe patients is attributed to platelet activation and platelet monocyte aggregation, leading to the induction of tissue factor (TF) [5]. Also, IL-1β is known to positively regulate TF expression. Taming the cytokine storm can be an effective management strategy and IL-1 signaling might play a crucial role in the pathogenesis of the disease. It could be initial necrosis liberating IL-1α, which drives amplifying inflammation loop eventually leading to inflammasome-dependent pyroptosis determining the severity of the disease. Anakinra (IL-1α and IL-1β blocker) has shown greatly reduced mortality, need for invasive mechanical ventilation, and bettering oxygenation status in severe COVID-19 patients in smaller clinical studies [1]. Hence, anakinra might be effective in managing the severe pandemic state; more so, it is a well-tolerated molecule with no adverse effects. Larger controlled clinical trials with anakinra are much warranted to test the efficacy unequivocally.

## Data Availability

Literature survey
